# Modeling Tree-like Heterophily on Symmetric Matrix Manifolds

**DOI:** 10.3390/e26050377

**Published:** 2024-04-29

**Authors:** Yang Wu, Liang Hu, Juncheng Hu

**Affiliations:** College of Computer Science and Technology, Jilin University, Changchun 130012, China; yangwu16@mails.jlu.edu.cn (Y.W.); hul@jlu.edu.cn (L.H.)

**Keywords:** graph neural networks, tree-like structure, symmetric matrix manifold, information propagation

## Abstract

Tree-like structures, characterized by hierarchical relationships and power-law distributions, are prevalent in a multitude of real-world networks, ranging from social networks to citation networks and protein–protein interaction networks. Recently, there has been significant interest in utilizing hyperbolic space to model these structures, owing to its capability to represent them with diminished distortions compared to flat Euclidean space. However, real-world networks often display a blend of flat, tree-like, and circular substructures, resulting in heterophily. To address this diversity of substructures, this study aims to investigate the reconstruction of graph neural networks on the symmetric manifold, which offers a comprehensive geometric space for more effective modeling of tree-like heterophily. To achieve this objective, we propose a graph convolutional neural network operating on the symmetric positive-definite matrix manifold, leveraging Riemannian metrics to facilitate the scheme of information propagation. Extensive experiments conducted on semi-supervised node classification tasks validate the superiority of the proposed approach, demonstrating that it outperforms comparative models based on Euclidean and hyperbolic geometries.

## 1. Introduction

The prevalence of hierarchical tree-like structures, characterized by power-law distributions, is a ubiquitous phenomenon observed across various real-world applications, encompassing domains from social networks [1,2] to data mining [3] and recommendation systems [4]. This pervasive structural pattern has garnered significant attention within the realm of computer science and network analysis due to its profound implications for comprehending network dynamics, functionality, and evolution [5,6,7].

In recent years, there has been a burgeoning interest among researchers in employing hyperbolic space modeling to elucidate tree structures. In contrast to the conventional Euclidean spaces characterized by zero curvature, hyperbolic spaces, endowed with negative curvature, offer a more nuanced measure of inter-nodal distances within a tree. Moreover, the intrinsic property of hyperbolic space to manifest exponential expansion aligns seamlessly with the exponential proliferation inherent in tree growth dynamics.

**Figure 1:**
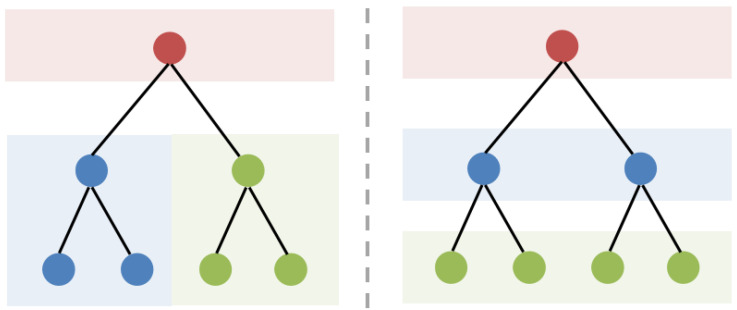
Illustration of homophily (**left**) and heterophily (**right**) derived from tree-like graphs. Colors denote node categories.

The complexities inherent in real-world networks often entail a broad spectrum of structural motifs, encompassing flat, tree-like, and circular substructures, thereby giving rise to heterophily within the network. Heterophily contrasts with homophily, where nodes sharing similar attributes tend to cluster together. As depicted in Figure 1, within the overarching tree-like structure, the diverse properties of local substructures yield a variety of graphs. The left graph shows cluster-forming sub-trees, reflecting homophily, while the right graph exhibits hierarchical sub-trees, indicative of heterophily. Hyperbolic spaces offer a nuanced depiction of hierarchical structures and exponential growth dynamics, whereas Euclidean spaces are valued for their simplicity and intuitive geometric properties. Regardless of whether one opts to model such networks within the framework of hyperbolic or Euclidean spaces, both approaches inevitably encounter challenges related to local distortion, resulting in the inaccurate modeling of distances between nodes.

**Figure 2:**
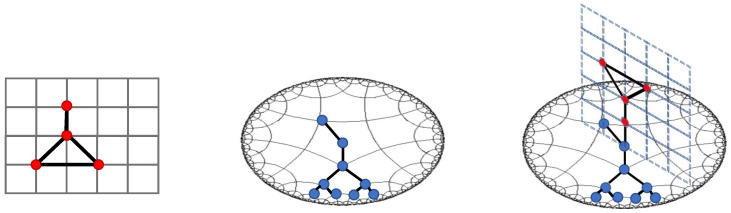
Illustration of graphs embedded in a continuous symmetric space with both flat and tree-like substructures.

To mitigate the limitations above, this study seeks to explore a more expressive space that could tolerate structural heterophily. The aim is to encode the information inherent in the graph topology into a continuous embedding space with less distortion, thus enhancing the performance of the downstream node classification task. From a geometric perspective, the quality of the embedding in geometric learning depends on the compatibility between the intrinsic graph structure and the embedding space. In light of this principle, we employ the Riemannian manifold of symmetric positive-definite matrices to embed node representations. As shown in Figure 2, symmetric spaces have a rich structure of totally geodesic subspaces, including flat (Euclidean) subspaces and tree-like (hyperbolic) subspaces, facilitating the representations of various substructures within a continuous space.

In Riemannian geometry, a Riemannian metric is a fundamental concept used to define distances, angles, and other geometric properties on smooth manifolds. Various Riemannian metrics have been proposed to guarantee the geometric properties of a symmetric positive-definite manifold (SPD), including the affine-invariant metric (AIM) [8], log-Euclidean metric (LEM) [9,10], and log-Cholesky metric (LCM) [11]. Equipped with these metrics, many Euclidean methods can be generalized into the domain of the Riemannian manifold.

In this study, we introduce a novel approach termed Riemannian graph convolutional neural network (RGCN) aimed at effectively capturing tree-like heterophily within graphs. RGCN operates on the Riemannian symmetric positive-definite matrix manifold and utilizes pullback techniques to generalize Riemannian metrics, such as LEM and LCM, to reconstruct key components of graph convolutional neural networks. In particular, the pullback technique first maps the embedding from the SPD manifold onto the tangent space, proceeds with the operations of information propagation, and ultimately pulls the resulting embeddings back to the SPD manifold. These information propagation components encompass feature transformation, neighborhood aggregation, and non-linear activation, as detailed in prior work [12]. Specifically, the integration of feature transformation and non-linear activation enriches the expressive capacity of the SPD neural network. Concurrently, the iterative process of neighborhood aggregation updates the node embeddings by transporting neighboring features across the graph topology. Our experimental results on semi-supervised node classification tasks substantiate the superiority of our proposed methodology, consistently surpassing comparative models grounded in Euclidean and hyperbolic geometries. The principal contributions of this research can be outlined as follows:

- Introduction of a graph convolutional neural network framework operating on the Riemannian symmetric positive-definite matrix manifold, facilitating graph embedding with reduced distortion and enhanced expressiveness.
- Development of a comprehensive scheme of information propagation on the symmetric positive-definite matrix manifold through the utilization of pullback techniques for the generalization of various Riemannian metrics.
- Extensive experimental evaluations showing the significant performance enhancements achieved by our proposed RGCN model compared to existing Euclidean and hyperbolic baselines in the context of semi-supervised node classification tasks.

The rest of this paper is organized as follows. In Section 2, we briefly survey the related works about GNNs and Riemannian manifolds of symmetric positive-definite matrices. Section 3 introduces some preliminaries. Section 4 presents the details of our proposed model. In Section 5, experimental results on eight benchmark datasets are shown and analyzed to highlight the benefits of our approach. Finally, we conclude the paper in Section 6.

## 2. Related Work

### 2.1. Graph Neural Networks

Contemporary graph neural network (GNN) models commonly embrace the message-passing paradigm [13] to encode node representations, demonstrating significant achievements across tasks such as node classification [12], link prediction [14], and graph classification [15]. Advancements in this domain are typically categorized into two primary branches: spectral approaches [16,17] and spatial approaches [12,18]. Spectral approaches leverage graph spectral theory to define graph convolutional operations. Taking inspiration from [19], which suggests approximating spectral filters via truncated Chebyshev polynomial expansions of the graph Laplacian, ChebNet [17] introduces *K*-localized convolutions, laying the groundwork for convolutional neural networks on graphs. Expanding upon this, graph convolutional network (GCN) [12] restricts the *K*-localized convolution to K=1, employing multiple layers to implement rich convolutional filter functions. To address both local and global consistency, deep graph convolutional neural network (DGCNN) [20] extends GCN by integrating a convolutional operation with a positive pointwise mutual information matrix. Conversely, spatial approaches directly aggregate neighborhood information around the central node. For example, GraphSAGE [12] introduces a versatile inductive framework that samples fixed-size local neighborhoods and aggregates their features using mean, long short-term memory (LSTM) or pooling mechanisms. Graph attention network (GAN) [21] enhances this aggregation process with attention mechanisms, assigning varying weights to aggregated neighborhoods through self-attention mechanisms. Despite the robust theoretical foundation of spectral-based GCNs, spatial-based GCNs demonstrate superior efficiency, generality, and adaptability. For deeper insights into graph neural networks, numerous comprehensive surveys are available [22,23].

Researchers have observed that numerous graphs, including social networks and biological networks, often manifest a pronounced hierarchical structure [24]. Krioukov et al. [25] emphasized that the strong clustering and power-law degree distribution properties in such graphs can be ascribed to a latent hierarchy. Recent investigations have underscored the remarkable representational efficacy of hyperbolic spaces in modeling underlying hierarchies across diverse domains, such as taxonomies [26,27], knowledge graphs [28,29], images [30], semantic classes [31], and actions [32], yielding promising outcomes. Liu et al. [33] and Chami et al. [34] have proposed hyperbolic graph convolutional networks (HGCNs), extending GCNs to hyperbolic spaces for capturing hierarchical structures in graphs. Recently, a series of GNNs have emerged in these spaces, executing graph convolution on various Riemannian manifolds to accommodate diverse graph structures, such as hyperbolic space on tree-like graphs [25], spherical space on spherical graphs [35], and their Cartesian products [36,37].

### 2.2. Riemannian Manifold of Symmetric Positive-Definite Matrices

The utilization of symmetric positive-definite (SPD) matrices for data representation has been a topic of extensive investigation, primarily leveraging covariance matrices to capture the statistical dependencies among Euclidean features [38,39]. Recent research endeavors have shifted towards the development of foundational components of neural networks within the covariance matrix space. This includes techniques for feature transformation, such as mapping Euclidean features to covariance matrices using geodesic Gaussian kernels [40], non-linear operations applied to the eigenvalues of covariance matrices [41], convolutional operations employing SPD filters [42], and the Frechét mean [43]. Furthermore, proposals for Riemannian recurrent networks [44] and Riemannian batch normalization [45] have been put forth. In comparison to these prior approaches, our proposal introduces an adaptive framework utilizing the pullback paradigm to construct the information propagation component with both LEM and LCM.

## 3. Preliminaries and Problem Definition

In this section, we initially introduce the preliminaries and notation essential for constructing an SPD embedding space. Subsequently, we define the problem of semi-supervised node classification on the SPD manifold.

### 3.1. Riemannian Manifold

A smooth manifold M extends the concept of a surface to higher dimensions. At each point x∈M, there is an associated tangent space $T_{x}M$, representing the first-order approximation of M around x, which is locally Euclidean. The Riemannian metric $g_{x}\left(\cdot ,\cdot \right):T_{x}M\times T_{x}M\to R$ defined on the tangent space $T_{x}M$ induces an inner product, enabling the derivation of geometric concepts. The pair (M,g) constitutes a Riemannian manifold. The transition between the tangent space and the manifold is facilitated by the exponential and logarithmic maps, denoted as $\exp_{x}\left(v\right):T_{x}M\to M$ and $\log_{x}\left(y\right):M\to T_{x}M$, respectively. Here, $\exp_{x}\left(v\right)$ projects the vector $v\in T_{x}M$ onto the manifold M at point x, while $\log_{x}\left(y\right)$ projects the vector y∈M back to the tangent space $T_{x}M$. For further elucidation, please consult the mathematical references [46].

### 3.2. Geometry of SPD Manifold

SPD matrices constitute a subset of the Euclidean space $R^{n(n+1)/2}$, and various well-established Riemannian metrics exist on the SPD manifold. Here, we briefly provide an overview of two such metrics, namely, LEM [9] and LCM [11]. The matrix logarithms $\log:S_{++}^{n}\to S^{n}$ and $\log^{lcm}:S_{++}^{n}\to {𝓛}^{n}$ are defined as follows:

(1) $$\log^{lem}\left(S\right)=U\ln\left(\Lambda \right)U^{⊤},$$

(2) $$\log^{lcm}\left(S\right)=\phi \left(L\left(S\right)\right),$$

where $S=U\Lambda U^{⊤}$ denotes the eigenvalue decomposition, L=L(S) represents the Cholesky decomposition, ϕ(L)=⌊L⌋+ln(D(L)) signifies a coordinate transformation from the ${𝓛}_{+}^{n}$ manifold onto the Euclidean space ${𝓛}^{n}$, ⌊L⌋ denotes the strictly lower triangular part of L, and D(L) represents the diagonal elements. It is noteworthy that, topologically, ${𝓛}^{n}≃S^{n}≃R^{n(n+1)/2}$, as their metric topology stems from the Euclidean metric tensor. Leveraging the matrix logarithm, Arsigny et al. [9] propose LEM via Lie group translation, while Lin et al. [11] introduce LCM based on the Cholesky logarithm, establishing an isometry between $S_{++}^{n}$ and ${𝓛}_{+}^{n}$. In this investigation, we posit that LEM and LCM are fundamentally analogous, reflecting a high-level mathematical abstraction.

The Riemannian metric and corresponding geodesic distance under the LEM are expressed as follows:

(3) $$g_{S}^{\mathrm{lem}}\left(V_{1},V_{2}\right)=g^{E}\left(\log_{*,S}^{\mathrm{lem}}\left(V_{1}\right),\log_{*,S}^{\mathrm{lem}}\left(V_{2}\right)\right),$$

(4) $$d^{\mathrm{lem}}\left(S_{1},S_{2}\right)={∥\log^{\mathrm{lem}}\left(S_{1}\right),\log^{\mathrm{lem}}\left(S_{2}\right)∥}_{F},$$

where $S\in S_{++}^{n}$, $V_{1},V_{2}\in T_{S}S_{++}^{n}$ are tangent vectors, $\log_{*,S}^{\mathrm{lem}}\left(\cdot \right)$ denotes the differential map of the matrix logarithm at S, $g^{E}$ represents the standard Euclidean metric tensor, and ${∥\cdot ∥}_{F}$ stands for the Frobenius norm.

Similarly, the Riemannian metric and geodesic distance under LCM are defined as

(5) $$g_{S}^{\mathrm{lcm}}\left(V_{1},V_{2}\right)={\tilde{g}}_{L}\left(L{\left(L^{-1}V_{1}L^{-⊤}\right)}_{\frac{1}{2}},L{\left(L^{-1}V_{2}L^{-⊤}\right)}_{\frac{1}{2}}\right),$$

(6) $$d^{\mathrm{lcm}}\left(S_{1},S_{2}\right)=\{∥\left\lfloor L_{1}\right\rfloor -\left\lfloor L_{2}\right\rfloor {∥}_{F}^{2}+∥\ln\left(D\left(L_{1}\right)\right)-\ln\left(D\left(L_{2}\right)\right){{∥}_{F}^{2}\}}^{\frac{1}{2}},$$

where $S\in S_{++}^{n}$, $V_{1},V_{2}\in T_{S}S_{++}^{n}$, $X_{\frac{1}{2}}=\left\lfloor X\right\rfloor +D\left(X\right)/2$, and ${\tilde{g}}_{L}\left(\cdot ,\cdot \right)$ denotes the Riemannian metric on ${𝓛}_{+}^{n}$, defined as

(7) $${\tilde{g}}_{L}\left(X_{1},X_{2}\right)=g^{E}\left(\left\lfloor X_{1}\right\rfloor ,\left\lfloor X_{2}\right\rfloor \right)+g^{E}\left(D{\left(L\right)}^{-1}\left(DX_{1}\right),D{\left(L\right)}^{-1}D(X_{2})\right).$$

### 3.3. Problem Definition

In this study, we delve into semi-supervised graph representation learning within the SPD space. For clarity and without loss of generality, we define a graph G=(V,E,X), where $V=\{v_{1},\cdots ,v_{n}\}$ represents the node set and $E=\{\left(v_{i},v_{j}\right)|v_{i},v_{j}\in V\}$ denotes the edge set. The edges are encapsulated in the adjacency matrix A, where $A_{ij}=1$ if $(v_{i},v_{j})\in E$ and 0 otherwise. Each node $v_{i}$ is characterized by a feature vector $x_{i}\in R^{d}$, and matrix $X\in R^{|V|\times d}$ represents the collective features of all nodes. We now formalize the problem at hand.

**Definition** **1** (Semi-supervised graph representation learning in the SPD space). *Given a graph G=(V,E,X), the objective of semi-supervised graph representation learning in the SPD space is to ascertain an encoding function Φ:V→Z that maps each node v to a vector z within an SPD space. This encoding should encapsulate the intrinsic complexity of the graph structure, leveraging information from a subset of labeled nodes to enable accurate label predictions for unlabeled nodes.*

## 4. SPD Graph Convolutional Networks

Our approach, RGCN, introduces an innovative graph neural network framework constructed on the SPD manifold. Drawing upon the foundation established by HGCN, we conduct graph convolution operations within the substituted Euclidean space and subsequently pull the embeddings back to the SPD manifold. Following the paradigm of GCN and HGNN architectures, RGCN comprises three essential components: feature transformation, neighborhood aggregation, and non-linear activation.

### 4.1. Mapping from Euclidean to SPD Spaces

RGCN initially projects input features onto the SPD manifold using the exp map. Let $x^{E}\in R$ represent input Euclidean features, which may be generated by pre-trained Euclidean neural networks. The objective is to devise a transformation that maps these Euclidean features to a point within the SPD space. To achieve this, we learn a linear map that converts the input Euclidean features into a vector of dimension n(n+1)/2, which is reshaped to form the lower triangle of an initially zero matrix $A\in R^{n\times n}$. Subsequently, we apply the exponential map to transition the coordinates from the substituted Euclidean space to the original manifold $S_{++}^{n}$. For instance, in the case of LEM, we define a symmetric matrix $U\in S^{n}$ such that $U=A+A^{⊤}$, followed by the exp map as the inverse of Equation (1):

(8) $$Z^{0}=\exp^{lem}\left(U\right);$$

whereas for LCM, we directly employ the exp map as the inverse map of Equation (2):

(9) $$Z^{0}=\exp^{lcm}\left(A\right)=S\left(\Phi \left(A\right)\right),$$

where S(·) represents the inverse of the Cholesky decomposition, Φ(L)=⌊L⌋+exp(D(L)) signifies a coordinate transformation from the Euclidean space ${𝓛}^{n}$ onto the ${𝓛}_{+}^{n}$ manifold. This one-time mapping process enables input features to operate within the SPD manifold seamlessly.

### 4.2. Feature Transformation

The feature transformation employed in the standard GCN is utilized to map the embedding space of one layer to the embedding space of the next layer, aiming to capture large neighborhood structures. In our approach, we aim to learn transformations of points on the SPD manifold. However, SPD space lacks the notion of a vector space structure. To address this, we extend the framework provided by HGCN and derive transformations within this space. The core concept is to leverage the matrix exponential (exp) and logarithm (log) maps, enabling us to perform Euclidean transformations using substituted Euclidean subspaces $S^{n}$ or ${𝓛}^{n}$. Assuming ***W*** is an $n^{\prime }\times n$ weight matrix, we define the SPD linear transformation as follows:

(10) $$W⊗Z:=\exp(W\log\left(Z\right)W^{⊤}),$$

where both the exp and log maps can be formulated using techniques such as the log-Euclidean metric (LEM) or log-Cholesky metric (LCM).

### 4.3. Neighborhood Aggregation

Neighborhood aggregation stands as a pivotal operation within GCNs, enabling the capture of intricate neighborhood structures and features. Let us consider that $x_{i}$ aggregates information from its neighbors $(x_{j})j\in N(i)$ with associated weights $(w_{j})j\in N(i)$. While mean aggregation in Euclidean GCNs computes the weighted average $\sum_{j\in N(i)}w_{j}x_{j}$, an analogous operation in hyperbolic space, known as the Fréchet mean, lacks a closed-form solution. To address this, we propose aggregation within substituted Euclidean subspaces $S^{n}$ or ${𝓛}^{n}$ employing an attention mechanism.

In GCNs, attention learns the significance of neighbors and aggregates their information based on their relevance to the central node. Yet, attention on Euclidean embeddings often overlooks the tree-like structure prevalent in many real-world graphs. Thus, we further propose an SPD attention-based aggregation operation. Given SPD embeddings $\left(Z_{i},Z_{j}\right)$, we initially map $Z_{i}$ and $Z_{j}$ to substituted Euclidean subspaces $S^{n}$ or ${𝓛}^{n}$ to compute attention weights $w_{ij}$ using concatenation and a Euclidean multi-layer perceptron (MLP). Subsequently, we propose SPD aggregation to update node embeddings as follows:

(11) $$w_{ij}={\mathrm{SOFTMAX}}_{j\in N(i)}\left(\mathrm{MLP}\left(log\left(Z_{i}\right)∥log\left(Z_{j}\right)\right)\right),$$

(12) $$\mathrm{AGG}\left(Z_{i}\right)=\exp\left(\sum_{j\in N(i)}w_{ij}\log\left(Z_{j}\right)\right).$$

Similar to Euclidean aggregation, RGCN employs a non-linear activation function, $\sigma^{S}(\cdot )$, to learn non-linear transformations. Specifically, RGCN applies the Euclidean non-linear activation in substituted Euclidean subspaces $S^{n}$ or ${𝓛}^{n}$, and then, maps back to the SPD manifold $S_{++}^{n}$:

(13) $$\sigma^{S}(Z)=\exp\left(\sigma^{E}\left(\log\left(Z\right)\right)\right).$$

It is worth noting that the exponential and logarithm maps are instantiated by both the log-Euclidean metric (LEM) and log-Cholesky metric (LCM).

### 4.4. RGCN Architecture

**Figure 3:**
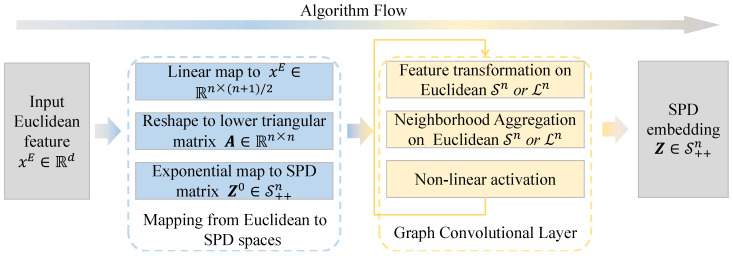
Schematic of RGCN.

Having introduced all the building blocks of RGCN, we now summarize the model architecture, as illustrated in Figure 3. Given a graph G=(V,E) and input Euclidean features ${\left(x^{E}\right)}_{i\in V}$, the first layer of RGCN maps from Euclidean to SPD space. RGCN then stacks multiple SPD graph convolution layers. At each layer HGCN transforms and aggregates neighbor’s embeddings in the substituted Euclidean subspaces. Hence, the information propagation in an RGCN layer is:

(14) $$\begin{array}{ccc} \; & H_{i}^{\ell }=W⊗Z_{i}^{\ell -1} & (\mathrm{feature}\;\mathrm{transformation})\\ \; & Y_{i}^{\ell }=\mathrm{AGG}\left(H_{i}^{\ell }\right) & (\mathrm{neighborhood}\;\mathrm{aggregation})\\ \; & Z_{i}^{\ell }=\sigma^{S}(Y_{i}^{\ell }) & (\mathrm{non-linear}\;\mathrm{activation}) \end{array}$$

SPD embeddings ${\left(Z\right)}_{i\in V}$ of the last RGCN layer can then be used to predict node labels. For the node classification task, we directly classify the nodes on the SPD manifold using the SPD multinomial logistic loss.

## 5. Experiments

In this section, we present our experimental evaluation to validate the effectiveness of the proposed method and analyze the results.

### 5.1. Experimental Setup

**Datasets.** Our evaluation employs several real-world graph datasets, encompassing two tree-like graphs labeled as Disease and Airport, five tree-like heterophily graphs (hyperlink networks of universities including Texas, Wisconsin, and Cornell, as well as webpage graphs discussing related topics such as Squirrel and Chameleon), and two benchmark homophily graphs (Cora, and PubMed). Gromov’s δ-hyperbolicity [34], an index derived from group theory, quantifies the tree-like structure of a graph. A lower δ value indicates a stronger tendency towards a tree-like structure, i.e., a hierarchical arrangement. Specifically, δ=0 represents a fully tree-like structure. The data’s statistics and hyperbolicity metrics are summarized in Table 1.

**Baselines.** We benchmark our proposed model against various baselines: (1) Shallow Euclidean models, specifically MLP; (2) Euclidean GNN models, comprising GCN [12], SGC [47], GAT [21], SAGE [48], GeomGCN [18], GCNII [49], and H_2_GCN [50]; and (3) hyperbolic GNN models, namely, HGCN [34], HAT [51], LGCN [52], and HyboNet [53]. Table 2 presents a comparative analysis of these models, delineating their respective capabilities in terms of global tree-likeness modeling, local heterophily perception, and interactional proficiency with neighboring information.

**Experimental Details.** We adhere to a consistent data splitting strategy employed in previous studies [18,34]. Specifically, nodes within the Disease category are partitioned into training (30%), validation (10%), and test (60%) sets. For other categories such as Texas, Wisconsin, Cornell, Squirrel, and Chameleon, the node distribution is set at 70%, 15%, and 15%, respectively. However, for Cora and PubMed, we utilize 20 labeled training examples per class. Our methodology closely mirrors the parameter configurations and optimization techniques outlined in the original works.

The implementation of the proposed RGCN is realized using PyTorch and PyTorch Geometric (PyG), a specialized deep learning library tailored for graph-structured data and built upon PyTorch. To ensure equitable model comparisons across datasets, we employ identical data splitting and 10-fold cross-validation procedures, reporting average F1 scores and standard deviation. Specifically, for RGCN, we report optimal results within LEM and LCM, adjusting the following hyperparameters: (1) hidden layer dimension (dim∈3,5,7,10,15), (2) number of propagation layers (layer∈2,3,4,5,6), (3) dropout rate (dropout∈0,0.1,0.5,0.7,0.9), (4) learning rate (lr∈0,0.005,0.01,0.05,0.1), and (5) weight decay ($\in 0,1\times 10^{-4},1\times 10^{-3},1\times 10^{-2},0.1$). RGCN employs early stopping with 100 epochs based on validation set performance. The experiments are conducted on an Intel(R) Xeon(R) Gold 5220 CPU @ 2.20 GHz, Quadro @ RTX 6000 hardware configuration.

### 5.2. Experimental Results

The proposed RGCN model is initially assessed in the context of node classification to gauge its discriminative capacity across tree-like and grid-like structures. Table 3 presents performance comparisons between different models, encompassing those operating within Euclidean and hyperbolic spaces. Notably, models leveraging hyperbolic geometry exhibit substantial performance gains over several comparative models, particularly evident in datasets resembling tree structures, such as the Disease dataset, exhibiting complete tree-like structures when δ=0. This underscores the efficacy of hyperbolic geometry in adeptly capturing hierarchical structures within graphs. As illustrated in the table, the proposed RGCN achieves peak performance on three out of four datasets, displaying slightly lower performance only on Cora, which tends towards Euclidean geometry. This underscores the effectiveness of symmetric positive-definite (SPD) geometry as an adaptive mixed space, encompassing both Euclidean and hyperbolic subspaces, for modeling intricate graphs comprising hierarchical and grid-like structures. Particularly noteworthy is the relative performance enhancements of 13.7% and 2.2% achieved by RGCN compared to methods solely based on Euclidean or hyperbolic geometry, respectively, on the real network Airport with δ=1. In summary, the utilization of SPD geometry by RGCN surpasses individual models grounded in hyperbolic and Euclidean geometries in modeling complex networks, with experimental outcomes validating the effective exploitation of SPD geometric properties in crafting neural network modules, thereby enhancing experimental performance.

Moreover, Table 4 presents the outcomes of graph neural network models predicated on Euclidean, hyperbolic, and symmetric positive-definite (SPD) geometries for node classification tasks on heterophily graphs. Notably, on intricate heterophily graphs, models grounded in hyperbolic geometry (e.g., HGCN and HyboNet) do not consistently surpass MLP. Specifically, hyperbolic models outperform the conventional homophily model GCN across nearly all five graph datasets; nevertheless, in comparison to heterophily models, they demonstrate superior performance solely on the Squirrel and Chameleon graphs, potentially attributable to disparities in specific graph structures.

Given that RGCN comprehensively harnesses the attributes of the SPD manifold, compatible with both Euclidean and hyperbolic geometries, it achieves the most remarkable classification results across all five datasets when compared to all comparative methodologies. In the outcomes pertaining to the initial three datasets, RGCN outshines the Euclidean heterophily graph model H_2_GCN, while in the results on the latter two datasets, RGCN’s performance also eclipses that of the hyperbolic model HyboNet. This unequivocally validates the geometric versatility of the SPD manifold and underscores the superiority of the proposed RGCN in modeling representation capabilities.

### 5.3. Analysis and Discussion

In this subsection, we analyze the sensitivity to hyperparameters regarding the embedding dimension and propagation layer.

**Figure 4:**
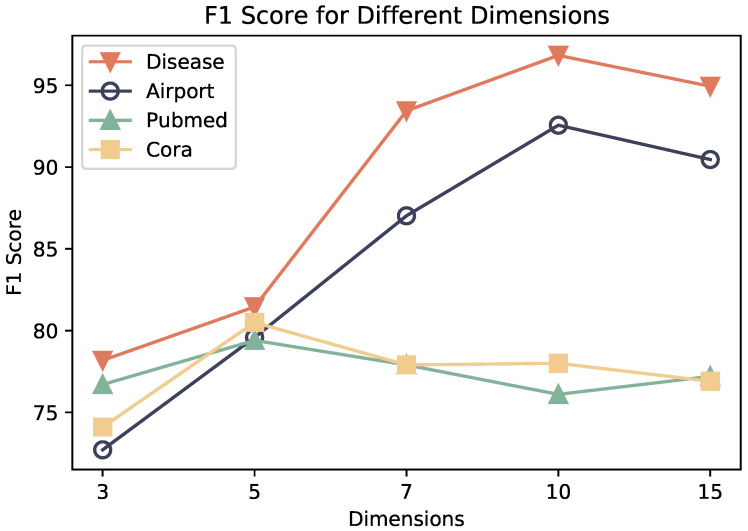
Classification results under different dimension settings.

For the hidden layer dimension, Figure 4 illustrates that on graphs biased towards hierarchical structures, such as Disease and Airport, optimal performance is attained at larger feature dimensions. Conversely, on graphs biased towards grid-like structures, such as Cora and PubMed, optimal performance is achieved at smaller dimensions, specifically at five dimensions. This variance in representation space dimensions due to geometric structural disparities aligns with the expectations of this study, indicating that the symmetric positive-definite (SPD) space encompasses both Euclidean and hyperbolic subspaces, enabling adaptive encoding of distinct spatial structures.

**Figure 5:**
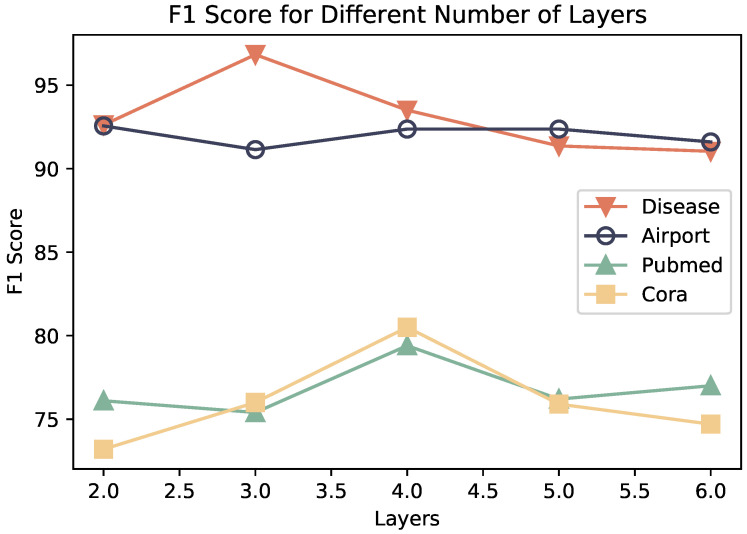
Classification results under different propagation layer settings.

Regarding the number of propagation layers, as depicted in Figure 5, the challenge of over-smoothing has long impeded graph neural networks from effectively capturing long-distance dependency relationships. Consequently, optimal performance of graph neural network models is typically achieved with fewer layers. The analysis of the number of propagation layers validates this observation. Although the optimal layer settings may vary due to different graph properties, optimal performance is generally attained within four layers, with a risk of over-smoothing when surpassing this threshold.

## 6. Conclusions

In this study, we systematically reconstructed the components of information propagation in classical Euclidean graph convectional networks, such as linear feature transformations, information aggregation, and non-linear activation functions, to symmetric manifold spaces, specifically symmetric positive-definite matrix spaces. By integrating Riemannian geometry with the log-Euclidean metric (LEM) and log-Cholesky metric (LCM) in pullback techniques, we develop a comprehensive scheme of information propagation on the symmetric positive-definite matrix manifold. Experimental results show that the proposed model outperforms its Euclidean and hyperbolic geometry counterparts on complex network data exhibiting implicit hierarchy. The efficacy of this approach further validates the applicability of deep learning to symmetric manifolds, offering a novel avenue for processing data with intricate structures. Although this study demonstrates the superiority of SPD manifolds over Euclidean and hyperbolic geometries for graph embedding, the neural network operations defined on SPD manifolds are computationally expensive. To enhance the scalability of SPD geometry on large-scale graph data, we will focus on the efficiency optimization of SPD neural networks in the future.

## Figures and Tables

**Table 1:** Data statistics.

Dataset	#Node	#Feature	#Class	#Edge	δ-Hyperbolicity
Disease	1044	1000	2	1043	0
Texas	183	1703	5	295	1
Wisconsin	251	1703	5	466	1
Cornell	183	1703	5	280	1
Squirrel	5201	-	5	198,493	1.5
Chameleon	2277	-	5	31,421	1.5
PubMed	19,717	500	3	88,651	3.5
Cora	2708	1433	7	5429	11

**Table 2:** Comparison of model capabilities regarding tree-like structure modeling, tree-like heterophily modeling, and neighbor interaction. A checkmark (✓) indicates the presence of the capability, while a cross (×) denotes its absence.

Model Type	Model	Tree	Heterophily	Neighbor
Shallow model	MLP	×	×	×
Euclidean GNNs	GCN [12]	×	×	✓
	SGC [47]	×	×	✓
	GAT [21]	×	×	✓
	SAGE [48]	×	×	✓
	GCNII [49]	×	×	✓
	GeomGCN [18]	×	✓	✓
	H_2_GCN [50]	×	✓	✓
Hyperbolic GNNs	HGCN [34]	✓	×	✓
	HAT [51]	✓	×	✓
	LGCN [52]	✓	×	✓
	HyboNet [53]	✓	×	✓
SPD model	RGCN	✓	✓	✓

**Table 3:** Node classification performance on various δ-hyperbolicity tree-like graphs (F1 score ± std).

Space	Model	Disease	Airport	PubMed	Cora
Euclidean	GCN [12]	69.7 ± 0.4	81.4 ± 0.6	78.1 ± 0.2	81.3 ± 0.3
	GAT [21]	70.4 ± 0.4	81.5 ± 0.3	79.0 ± 0.3	83.0 ± 0.7
	SAGE [48]	69.1 ± 0.6	82.1 ± 0.5	77.4 ± 2.2	77.9 ± 2.4
	SGC [47]	69.5 ± 0.2	80.6 ± 0.1	78.9 ± 0.0	81.0 ± 0.1
Hyperbolic	HGCN [34]	82.8 ± 0.8	90.6 ± 0.2	78.4 ± 0.4	81.3 ± 0.6
	HAT [51]	83.6 ± 0.9	–	78.6 ± 0.5	83.1 ± 0.6
	LGCN [52]	84.4 ± 0.8	90.9 ± 1.7	78.6 ± 0.7	**83.3 ± 0.7**
	HyboNet [53]	96.0 ± 1.0	90.9 ± 1.4	78.0 ± 1.0	80.2 ± 1.3
SPD	RGCN	**96.9 ± 0.9**	**92.6 ± 1.8**	**79.4 ± 1.2**	80.5 ± 1.5

**Table 4:** Node classification performance on heterophily graphs (F1 score ± std).

Dataset	Texas	Wisconsin	Cornell	Squirrel	Chameleon
MLP	80.8 ± 4.7	85.2 ± 3.3	81.9 ± 6.4	63.6 ± 2.1	72.8 ± 1.5
GCN [12]	55.1 ± 5.1	51.7 ± 3.0	60.5 ± 5.3	38.2 ± 1.6	60.4 ± 2.1
SAGE [48]	82.4 ± 6.1	81.1 ± 5.5	75.9 ± 5.0	41.6 ± 0.7	58.7 ± 1.6
GeomGCN [18]	66.7 ± 2.7	64.5 ± 3.6	60.5 ± 3.6	38.1 ± 0.9	60.0 ± 2.8
GCNII [49]	77.5 ± 3.8	80.3 ± 3.4	77.8 ± 3.7	56.6 ± 2.1	67.3 ± 2.4
H_2_GCN [50]	84.8 ± 7.2	86.6 ± 4.6	82.7 ± 5.2	51.0 ± 4.2	69.5 ± 1.8
HGCN [34]	70.1 ± 3.3	83.2 ± 4.5	79.4 ± 4.4	62.3 ± 1.5	74.9 ± 1.5
HyboNet [53]	72.2 ± 4.9	86.5 ± 4.5	77.2 ± 4.7	69.1 ± 1.6	78.7 ± 0.9
RGCN	**89.9 ± 6.6**	**88.7 ± 3.8**	**85.9 ± 5.1**	**75.3 ± 1.4**	**80.3 ± 0.6**

## Data Availability

Data are publicly available at https://github.com/HazyResearch/hgcn, (accessed on 17 February 2024).

## References

[1] Adcock A.B., Sullivan B.D., Mahoney M.W. (2013). Tree-like structure in large social and information networks. Proceedings of the 2013 IEEE 13th International Conference on Data Mining.

[2] Khrennikov A., Oleschko K. (2020). An ultrametric random walk model for disease spread taking into account social clustering of the population. Entropy.

[3] Hu X., Chen H., Chen H., Li X., Zhang J., Liu S. (2023). Mining Mobile Network Fraudsters with Augmented Graph Neural Networks. Entropy.

[4] Zhang X., Zhou Y., Wang J., Lu X. (2021). Personal interest attention graph neural networks for session-based recommendation. Entropy.

[5] Khrennikov A., Oleschko K., Correa Lopez M.d.J. (2016). Modeling fluid’s dynamics with master equations in ultrametric spaces representing the treelike structure of capillary networks. Entropy.

[6] Abu-Ata M., Dragan F.F. (2016). Metric tree-like structures in real-world networks: An empirical study. Networks.

[7] Xi Y., Cui X. (2023). Identifying Influential Nodes in Complex Networks Based on Information Entropy and Relationship Strength. Entropy.

[8] Pennec X., Fillard P., Ayache N. (2006). A Riemannian framework for tensor computing. Int. J. Comput. Vis..

[9] Arsigny V., Fillard P., Pennec X., Ayache N. (2005). Fast and Simple Computations on Tensors with Log-Euclidean Metrics. Ph.D. Thesis.

[10] Arsigny V., Fillard P., Pennec X., Ayache N. (2007). Geometric means in a novel vector space structure on symmetric positive-definite matrices. SIAM J. Matrix Anal. Appl..

[11] Lin Z. (2019). Riemannian geometry of symmetric positive definite matrices via Cholesky decomposition. SIAM J. Matrix Anal. Appl..

[12] Kipf T.N., Welling M. Semi-supervised classification with graph convolutional networks. Proceedings of the International Conference on Learning Representation.

[13] Gilmer J., Schoenholz S.S., Riley P.F., Vinyals O., Dahl G.E. Neural message passing for quantum chemistry. Proceedings of the International Conference on Machine Learning, PMLR.

[14] Zhang M., Chen Y. Link prediction based on graph neural networks. Proceedings of the Advances in Neural Information Processing Systems.

[15] Xu K., Hu W., Leskovec J., Jegelka S. How Powerful are Graph Neural Networks?. Proceedings of the International Conference on Learning Representations.

[16] Bruna J., Zaremba W., Szlam A., Lecun Y. Spectral networks and locally connected networks on graphs. Proceedings of the International Conference on Learning Representations (ICLR2014), CBLS.

[17] Defferrard M., Bresson X., Vandergheynst P. Convolutional neural networks on graphs with fast localized spectral filtering. Proceedings of the Advances in Neural Information Processing Systems.

[18] Pei H., Wei B., Chang K.C.C., Lei Y., Yang B. Geom-GCN: Geometric Graph Convolutional Networks. Proceedings of the International Conference on Learning Representations.

[19] Hammond D.K., Vandergheynst P., Gribonval R. (2011). Wavelets on graphs via spectral graph theory. Appl. Comput. Harmon. Anal..

[20] Zhang M., Cui Z., Neumann M., Chen Y. An end-to-end deep learning architecture for graph classification. Proceedings of the AAAI Conference on Artificial Intelligence.

[21] Veličković P., Cucurull G., Casanova A., Romero A., Lio P., Bengio Y. Graph attention networks. Proceedings of the International Conference on Learning Representations.

[22] Zhang Z., Cui P., Zhu W. (2020). Deep learning on graphs: A survey. IEEE Trans. Knowl. Data Eng..

[23] Peng W., Varanka T., Mostafa A., Shi H., Zhao G. (2021). Hyperbolic deep neural networks: A survey. IEEE Trans. Pattern Anal. Mach. Intell..

[24] Papadopoulos F., Kitsak M., Serrano M.Á., Boguná M., Krioukov D. (2012). Popularity versus similarity in growing networks. Nature.

[25] Krioukov D., Papadopoulos F., Kitsak M., Vahdat A., Boguná M. (2010). Hyperbolic geometry of complex networks. Phys. Rev. E.

[26] Nickel M., Kiela D. Poincaré embeddings for learning hierarchical representations. Proceedings of the Advances in Neural Information Processing Systems.

[27] Nickel M., Kiela D. Learning continuous hierarchies in the lorentz model of hyperbolic geometry. Proceedings of the International Conference on Machine Learning, PMLR.

[28] Sun Z., Chen M., Hu W., Wang C., Dai J., Zhang W. Knowledge Association with Hyperbolic Knowledge Graph Embeddings. Proceedings of the 2020 Conference on Empirical Methods in Natural Language Processing (EMNLP).

[29] Yang M., Zhou M., Pan L., King I. *κ*hgcn: Tree-likeness modeling via continuous and discrete curvature learning. Proceedings of the 29th ACM SIGKDD Conference on Knowledge Discovery and Data Mining.

[30] Khrulkov V., Mirvakhabova L., Ustinova E., Oseledets I., Lempitsky V. Hyperbolic image embeddings. Proceedings of the IEEE/CVF Conference on Computer Vision and Pattern Recognition.

[31] Liu S., Chen J., Pan L., Ngo C.W., Chua T.S., Jiang Y.G. Hyperbolic visual embedding learning for zero-shot recognition. Proceedings of the IEEE/CVF Conference on Computer Vision and Pattern Recognition.

[32] Zhang T., Zheng W., Cui Z., Zong Y., Li C., Zhou X., Yang J. (2020). Deep manifold-to-manifold transforming network for skeleton-based action recognition. IEEE Trans. Multimed..

[33] Liu Q., Nickel M., Kiela D. Hyperbolic graph neural networks. Proceedings of the Advances in Neural Information Processing Systems.

[34] Chami I., Ying Z., Ré C., Leskovec J. Hyperbolic graph convolutional neural networks. Proceedings of the Advances in Neural Information Processing Systems.

[35] Liu W., Wen Y., Yu Z., Li M., Raj B., Song L. Sphereface: Deep hypersphere embedding for face recognition. Proceedings of the IEEE Conference on Computer Vision and Pattern Recognition.

[36] de Ocáriz Borde H.S., Kazi A., Barbero F., Lio P. Latent graph inference using product manifolds. Proceedings of the Eleventh International Conference on Learning Representations.

[37] Sun L., Zhang Z., Ye J., Peng H., Zhang J., Su S., Philip S.Y. (2022). A self-supervised mixed-curvature graph neural network. Proc. AAAI Conf. Artif. Intell..

[38] Dong Z., Jia S., Zhang C., Pei M., Wu Y. Deep manifold learning of symmetric positive definite matrices with application to face recognition. Proceedings of the AAAI Conference on Artificial Intelligence.

[39] Gao Z., Wu Y., Bu X., Yu T., Yuan J., Jia Y. (2019). Learning a robust representation via a deep network on symmetric positive definite manifolds. Pattern Recognit..

[40] Brooks D.A., Schwander O., Barbaresco F., Schneider J.Y., Cord M. (2019). Exploring complex time-series representations for Riemannian machine learning of radar data. Proceedings of the ICASSP 2019–2019 IEEE International Conference on Acoustics, Speech and Signal Processing (ICASSP).

[41] Huang Z., Van Gool L. A riemannian network for spd matrix learning. Proceedings of the AAAI Conference on Artificial Intelligence.

[42] Zhang T., Zheng W., Cui Z., Li C. (2018). Deep manifold-to-manifold transforming network. Proceedings of the 2018 25th IEEE International Conference on Image Processing (ICIP).

[43] Chakraborty R., Bouza J., Manton J.H., Vemuri B.C. (2020). Manifoldnet: A deep neural network for manifold-valued data with applications. IEEE Trans. Pattern Anal. Mach. Intell..

[44] Chakraborty R., Yang C.H., Zhen X., Banerjee M., Archer D., Vaillancourt D., Singh V., Vemuri B. A statistical recurrent model on the manifold of symmetric positive definite matrices. Proceedings of the Advances in Neural Information Processing Systems.

[45] Brooks D., Schwander O., Barbaresco F., Schneider J.Y., Cord M. Riemannian batch normalization for SPD neural networks. Proceedings of the Advances in Neural Information Processing Systems.

[46] Spivak M. (1979). A Comprehensive Introduction to Differential Geometry, Publish or Perish.

[47] Wu F., Souza A., Zhang T., Fifty C., Yu T., Weinberger K. Simplifying graph convolutional networks. Proceedings of the International Conference on Machine Learning, PMLR.

[48] Hamilton W., Ying Z., Leskovec J. Inductive representation learning on large graphs. Proceedings of the Advances in Neural Information Processing Systems.

[49] Chen M., Wei Z., Huang Z., Ding B., Li Y. Simple and deep graph convolutional networks. Proceedings of the International Conference on Machine Learning, PMLR.

[50] Zhu J., Yan Y., Zhao L., Heimann M., Akoglu L., Koutra D. (2020). Beyond homophily in graph neural networks: Current limitations and effective designs. Adv. Neural Inf. Process. Syst..

[51] Zhang Y., Wang X., Shi C., Jiang X., Ye Y. (2021). Hyperbolic graph attention network. IEEE Trans. Big Data.

[52] Zhang Y., Wang X., Shi C., Liu N., Song G. Lorentzian graph convolutional networks. Proceedings of the Web Conference 2021.

[53] Chen W., Han X., Lin Y., Zhao H., Liu Z., Li P., Sun M., Zhou J. Fully Hyperbolic Neural Networks. Proceedings of the 60th Annual Meeting of the Association for Computational Linguistics.

